# Effect of Health Insurance Policy on the Health Outcomes of the Middle-Aged and Elderly: Progress Toward Universal Health Coverage

**DOI:** 10.3389/fpubh.2022.889377

**Published:** 2022-07-22

**Authors:** Xiaojing Fan, Min Su, Yaxin Zhao, Yafei Si, Duolao Wang

**Affiliations:** ^1^School of Public Policy and Administration, Xi'an Jiaotong University, Xi'an, China; ^2^School of Public Administration, Inner Mongolia University, Hohhot, China; ^3^School of Public Health, Xi'an Jiaotong University Health Science Centre, Xi'an, China; ^4^School of Risk and Actuarial Studies and Centre of Excellence in Population Aging Research (CEPAR), University of New South Wales, Kensington, NSW, Australia; ^5^Department of Clinical Sciences, Liverpool School of Tropical Medicine, Liverpool, United Kingdom

**Keywords:** health policy, chronic diseases, universal health coverage, CHARLS, middle-aged and elderly

## Abstract

This population-based study aims to explore the effect of the integration of the Urban and Rural Residents' Basic Medical Insurance (URRBMI) policy on the health outcomes of the middle-aged and elderly. A total of 13,360 participants in 2011 and 15,082 participants in 2018 were drawn from the China Health and Retirement Longitudinal Study. Health outcomes were evaluated using the prevalence of chronic diseases. A generalized linear mixed model was used to analyze the effect of the URRBMI policy on the prevalence of chronic disease. Prior to the introduction of the URRBMI policy, 67.09% of the rural participants and 73.00% of the urban participants had chronic diseases; after the policy's implementation, 43.66% of the rural participants and 45.48% of the urban participants had chronic diseases. When adjusting for the confounding factors, the generalized linear mixed model showed that the risk of having a chronic disease decreased by 81% [odds ratio (OR) = 0.19; 95% confidence interval (CI): 0.16, 0.23] after the introduction of the policy in the urban participants; in the rural participants, the risk of having a chronic disease was 30% lower (OR = 0.70; 95% CI: 0.60, 0.82) than the risk in the urban participants before the policy and 84% lower (OR = 0.16; 95% CI: 0.14, 0.19) after the implementation of the policy; the differences in the ORs decreased from 0.30 prior to the policy to 0.03 after the policy had been introduced between rural and urban participants when adjusting for the influence of socioeconomic factors on chronic diseases. This study provides evidence of the positive effects of the URRBMI policy on improving the rural population's health outcomes and reducing the gap in health outcomes between rural and urban populations, indicating that the implementation of the URRBMI policy has promoted the coverage of universal health.

## Introduction

Universal health coverage (UHC) is an important topic for the United Nations Sustainable Development Goals (1–3). The World Health Organization has used UHC as the theme of the World Health Report twice in a row (4). The 2016 annual report on world health statistics pointed out that achieving UHC has become an important goal and priority for the development of an international health system.

In 2003 and 2007, China established the New Rural Cooperative Medical Scheme (NCMS) and the Urban Residents' Basic Medical Insurance (URBMI) policy for rural and urban residents, respectively, in order to achieve UHC (5). The establishment of the NCMS and URBMI has played an important role in improving the basic medical security system for all residents, meeting the basic medical insurance and improving the health of residents (6–9). However, with the rapid economic and social development, negative effects of the urban–rural division of the two systems began to emerge, with problems such as large urban–rural disparities, poor transfer of medical insurance relationships, coverage overlaps, inconsistent settlement standards, and inconvenient reimbursement (10–12). In order to solve these problems and to advance the development of UHC, some provinces started to integrate rural and urban residents' medical insurance on a trial basis from 2010 onward. In 2016, the State Council of China issued the “Opinion on the Integration of Basic Medical Insurance Systems between Urban and Rural Residents” report on January 12, clearly proposing to integrate the NCMS and URBMI into the Urban and Rural Residents' Basic Medical Insurance (URRBMI) policy in other provinces based on the experience with the NCMS, URBMI, and trial provinces (13). URRBMI is of great significance for promoting social justice, for coordinated economic and social development in urban and rural areas, and for building a moderately prosperous society in China. Therefore, it is hypothesized that the health outcomes in rural areas would be increased after implementing the URRBMI policy.

The China Pension Financial Development Report (2016) stated that China's elderly population (over 65 years of age) will increase from 280 million (20.2%) in 2030 to 400 million (27.2%) in 2055 (14). The elderly comprises an important group in the utilization and protection of medical services in the context of their aging trend, their decreasing financial ability, and their actual situation of increasing medical expenses, which could lead to great financial pressure on elderly families. The issue of whether the URRBMI policy ensures a healthy later life for the elderly and reduces the financial pressure of medical treatment has received continuous attention from scholars across China. Recently, some scholars conducted extensive research on the impact of the URRBMI policy on residents' health from different perspectives. Zhu et al. concluded that the scale of financing for the URRBMI policy is insufficient for the increasing demands for medical services from the insured based on the data from the China Health Statistics Yearbook (11). Yang et al.'s study indicated that individuals received more inpatient service benefits after the URRBMI policy was implemented based on the data from the Fifth National Health Services Survey in 2013 (15). Liu et al.'s study showed that the gap between urban and rural areas in terms of receiving medical services and medical insurance narrowed after implementation of the URRBMI policy based on data from the primary medical institutions in 2011 in Baoji, Shaanxi (16). In summary, extensive research has been conducted on the correlation between health insurance and residents' health status from different perspectives, thereby achieving important progress and results and providing a rich platform for the empirical analysis and theoretical research of this study.

UHC is an ambitious goal in health service with sustainable development goal even during the COVID-19 Pandemic (4, 17–19). The lessons currently being learned from the COVID-19 pandemic in various countries around the world demonstrate the need to invest in health for all. Stable, equitable, prosperous and peaceful societies and economies is only possible when no one is left behind. In this study, two waves of China Health and Retirement Longitudinal study (CHARLS) in 2011 and 2018 based on face-to-face interviews were used first to explore whether the URRBMI policy has had a positive effect on improving the health outcomes of the middle-aged and elderly in rural areas in China, as well as whether the specific magnitude of the prevalence of chronic diseases has decreased. Finally, we drew preliminary conclusions about whether the Chinese health insurance policy is progressing in the right direction toward UHC.

## Methods

### Data and Sample

The data used in this study were from the 2011 and 2018 CHARLS before and after the introduction of China's URRBMI policy. The reason is as follows: As only some provinces have been implementing the URRBMI policy since around 2010, the pre-policy data were selected from the 2011 CHARLS; as most provinces have been implementing the URRBMI policy since the end of 2016, the post-policy data were selected from the 2018 CHARLS (a timeline of the policy implementation in each province is shown in Table 1). CHARLS is hosted by the National Development Research Institute of Peking University and is jointly implemented by the Chinese Social Science Research Center of Peking University and the University Youth League Committee (20). The CHARLS utilizes a multi-stage probability proportional scale sampling method to randomly select Chinese middle-aged and elderly people and their spouses from 150 counties and 450 communities (villages) across 28 provinces as respondents (21). The specific sampling process is as follows: firstly, all regions in China (excluding Tibet) were ranked according to GDP per capita, from which 150 county-level units (urban areas or rural counties) were selected at regular intervals. Secondly, three primary sampling units (PSUs) within each county were randomly selected using probability proportional to size sampling (PPS). The primary sampling units consisted of rural villages and urban communities, which are the lowest administrative level units of government. Thirdly, the households in each PSU were sampled with the help of CHARLS.GIS software. Questionnaires and medical examinations are conducted by face-to-face household surveys, which include information on demographics, physical examination, biochemical tests, socioeconomic status, health status and functioning, health care and insurance (the data and questionnaire are available at http://charls.pku.edu.cn/).

**Table 1:** The basic situation of urban and rural residents' basic medical insurance in China.

**Province**	**Name of policy**	**Year for implementation**
Tianjin	Regulations for integration of urban and rural residents' basic medical insurance in Tianjin	2010
Ningxia	Opinions on the coordination of integration of urban and rural residents' basic medical insurance	2010
Chongqing	Chongqing municipal cooperative medical insurance for urban and rural residents	2013
Shandong	Implementation plan of integration of urban and rural residents' basic medical insurance in Shandong province	2014
Shanghai	Integration of urban and rural residents' basic medical insurance in Shanghai	2016
Qinghai	The People' s Government for Qinghai Province on the issuance of the implementation plan for the provincial-level integration of urban and rural residents' basic medical insurance	2016
Guangdong	The People' s Government for Guangdong Province forwarded the “Opinions of the State Council on integration of urban and rural residents' basic medical insurance” notice	2016
Hebei	Opinions on the implementation of integration of urban and rural residents' basic medical insurance	2016
Hubei	Work plan for integration of urban and rural residents' basic medical insurance	2016
Inner Mongolia	Work plan for integration of urban and rural residents' basic medical insurance	2016
Jiangxi	Jiangxi issues implementation opinions on integration of urban and rural residents' basic medical insurance	2016
Xinjiang	Notice on the issuance of the implementation opinions on integration of urban and rural residents' basic medical insurance in Xinjiang Uygur Autonomous Region	2016
Beijing	Work Plan for integration of urban and rural residents' basic medical insurance in Beijing	2016
Yunnan	Implementation of integration of urban and rural residents' basic medical insurance in the People' s Government for Yunnan province	2016
Shaanxi	Notice on accelerating the implementation of a unified integration of urban and rural residents' basic medical insurance	2016
Zhejiang	A number of views of the People' s Government for Zhejiang province on the in-depth promotion of the integration of urban and rural residents' basic medical insurance	2016
Guangxi	Implementation opinions on the integration of urban and rural residents' basic medical insurance in the People' s government of Guangxi Zhuang Autonomous Region	2016
Shanxi	Implementation opinions on the integration of urban and rural residents' basic medical insurance in the People' s government of Shanxi Province	2016
Gansu	Issuance of integration of urban and rural residents' basic medical insurance in the People' s government of Gansu Province	2016
Henan	General Office of Henan Provincial People's Government on triggering the implementation of integration of urban and rural residents' basic medical insurance in Henan Province (for trial implementation)	2016
Heilongjiang	Integration of urban and rural residents' basic medical insurance in the People' s government of Heilongjiang Province	2016
Hunan	Implementation measures of integration of urban and rural residents' basic medical insurance in Hunan Province	2016
Sichuan	Sichuan issued on the implementation of the integration of urban and rural residents' basic medical insurance	2016
Jilin	Jilin issued on the implementation of the integration of urban and rural residents' basic medical insurance	2016
Hainan	Notice of Hainan provincial people's government on the issuance of the implementation plan for the integration of urban and rural residents' basic medical insurance in Hainan Province	2016
Jiangsu	Jiangsu provincial people's government on the implementation of the integration of urban and rural residents' basic medical insurance	2016
Guizhou	Notice of the provincial people's government on the issuance of the implementation plan for the integration of urban and rural residents' basic medical insurance in Guizhou Province	2016
Anhui	Anhui provincial people's government on the implementation of the integration of urban and rural residents' basic medical insurance	2016

**Figure 1:**
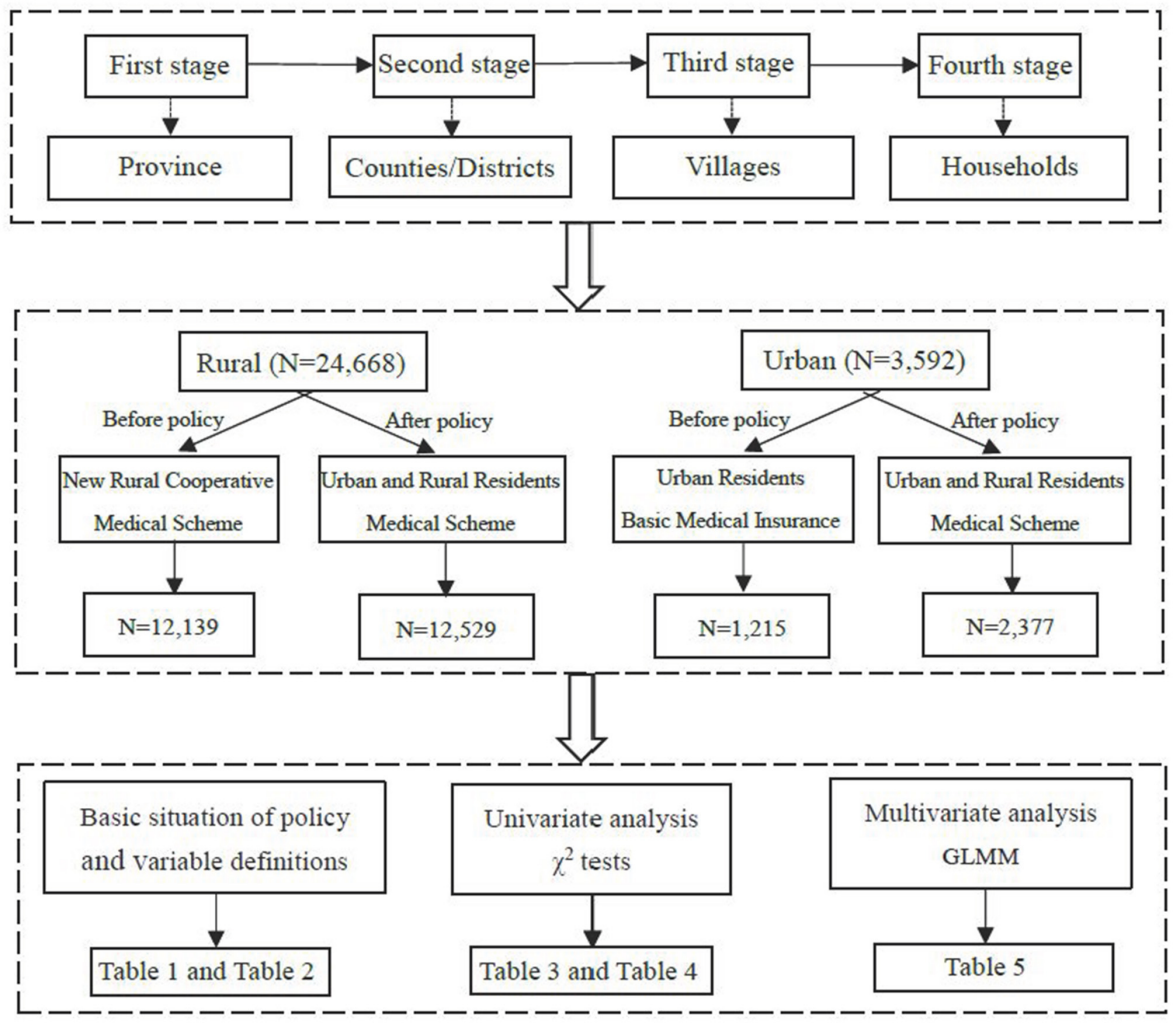
Flowchart on the sample selecting process and analysis framework.

A total of 17,705 household members in 2011 and 19,816 household members in 2018 were surveyed. The inclusion and exclusion criteria for this study were as follows: (1) informed consent signed by the study participants; (2) only participants who had experienced the NCMS and URBMI were selected as the sampling unit of interest in 2011; (3) in 2018, participants who had experienced the URRBMI were selected, although participants who had experienced NCMS or URBMI were also selected considering that some participants do not know the name of the integrated medical insurance. Finally, data from 13,360 participants in 2011 and 15,082 participants in 2018 were utilized for analysis. The detailed sample selecting process and the analysis framework are shown in Figure 1.

### Dependent Variable

This study used diagnosed chronic diseases or not to conceptualize health outcomes. This variable was based on respondents' reports on the following item: “Have you been diagnosed with [conditions listed below…] by a doctor?” The CHARLS questionnaire examined 14 chronic diseases: Hypertension, dyslipidemia, diabetes or elevated blood sugar, malignancies such as cancer (excluding mild skin cancer), chronic lung disease, liver disease, heart disease, stroke, kidney disease, stomach disease or digestive system disease, emotional and mental illness, memory-related disorders, arthritis or rheumatism, and asthma. The dependent variable was a binary variable, whereby 0 denoted “having no chronic disease” and 1 denoted “having one of these chronic diseases.” The prevalence of chronic diseases was calculated by the number of participants with chronic diseases over the total number of participants.

### Independent Variables

The core independent variable was the URRBMI policy. According to the policy, in which only rural participants changed from the NCMS to the URRBMI policy and the level of medical reimbursement improved, rural participants were selected to be the intervention group. For urban participants, although their health insurance name changed from URBMI to URRBMI, the level of medical reimbursement remained the same as the URBMI policy, making the urban participants an excellent control for this study. It was hypothesized that the prevalence of chronic diseases in rural areas would have decreased after implementing the URRBMI policy.

The control independent variables included the participants' sex, age, education, economic and living status, sleeping hours, napping or not, smoking status, alcohol consumption, disability and body pain or not. The participants' economic status was measured by household income per capita; it was used as a continuous variable, calculated as the sum of personal assets, household assets, personal income and income from other household members, household income from agriculture, household income from self-employment, and household income from transfers divided by the total number of family members in the household. It was further divided into three groups.

### Data Analysis

Ahead of inferential statistics, the characteristics of the sample were briefly described, followed by a Chi-squared test to compare the basic characteristics and health outcomes between the rural and urban participants for the years of 2011 and 2018. After this, a generalized linear mixed model (GLMM) was employed to analyze the effect of the URRBMI policy on health outcomes after controlling for a number of confounding factors. These factors were selected based on previous studies but constrained by the variables collected in the survey (22). Odds ratios (ORs) with 95% confidence intervals (CIs) were derived from the GLMM. The characteristics of all of the variables used are shown in Table 2. The variables policy, type of residence, and interaction between them were specified as fixed effects, and the community where they lived as a random effect. The participants' sex, age, education, economic status, living status, sleeping hours, napping or not, smoking status, alcohol consumption, disability, and pain or not were taken as covariates.

**Table 2:** Variable definitions and summary statistics.

**Variables**	**Group**	**Before policy (*****n*** **=** **13,360)**		**After policy (*****n*** **=** **15,082)**	
		**Rural**	**Urban**	**Rural**	**Urban**
**Control variables**					
**Demographics**					
Sex	Male	5,747 (47.38)	543 (44.69)	5,776 (46.10)	1,009 (42.45)
	Female	6,382 (52.62)	672 (55.31)	6,753 (53.90)	1,368 (57.55)
Age (years)	45–50	2,735 (22.53)	275 (22.63)	1,487 (11.87)	352 (14.81)
	51–60	4,523 (37.26)	451 (37.12)	4,029 (32.16)	914 (38.45)
	61–70	3,205 (26.40)	316 (26.01)	4,283 (34.18)	718 (30.21)
	≥71	1,676 (13.81)	173 (14.24)	2,730(21.79)	393 (16.53)
Education	Illiterate	3,982 (32.84)	198 (16.31)	3,593 (28.68)	343 (14.43)
	≤ Elementary school	5,204 (42.92)	460 (37.89)	5,951 (47.50)	1,015 (42.70)
	≥Middle school	2,940 (24.25)	556 (45.80)	2,985 (23.82)	1,019 (42.87)
Economic status	Low	932(7.85)	108 (9.25)	5,173 (41.29)	535 (22.51)
	Middle	6,116 (51.54)	415 (35.56)	6,076 (48.50)	1,366 (57.47)
	High	4,818 (40.60)	644 (55.18)	1,279 (10.21)	476 (20.03)
**Life style**					
Living status	Live with others	9,663 (79.60)	989 (81.40)	9,797 (78.19)	1,805 (75.94)
	Live alone	2,476 (20.40)	226 (18.60)	2,732 (21.81)	572 (24.06)
Sleeping hours	7–8 h	4,445 (36.62)	481 (39.59)	4,402 (35.13)	889 (37.40)
	≤ 6 h	5,667 (46.68)	562 (46.26)	6,752 (53.89)	1,323 (55.66)
	>8 h	2,027 (16.70)	172 (14.16)	1,375 (10.97)	165 (6.94)
Nap time (min)	No	5,481 (45.15)	505 (41.56)	5,075 (40.51)	845 (35.55)
	Yes	6,658 (54.85)	710 (58.44)	7,454 (59.49)	1,532 (64.45)
Smoking status	No	7,229 (59.60)	773(63.67)	7,212 (94.67)	1,472 (91.54)
	Yes	4,900 (40.40)	441(36.33)	406 (5.33)	136 (8.46)
Alcohol consumption	No	8,123 (66.99)	845 (69.60)	8,573 (68.44)	1,609 (67.72)
	Yes	4,002 (33.01)	369 (30.40)	3,954 (31.56)	767 (32.28)
**Health status**					
Disability	No	9,768 (80.59)	1,000 (82.37)	7,418 (81.39)	1,640 (86.27)
	Yes	2,353 (19.41)	214 (17.63)	1,696 (18.61)	261 (13.73)
Body pain	No	7,868 (64.82)	864 (71.11)	4,838 (38.61)	1,044 (43.92)
	Yes	4,271 (35.18)	351 (28.89)	7,691 (61.39)	1,333 (56.08)
**Dependent variables**					
Chronic disease					
	No	3,995 (32.91)	328 (27.00)	7,059 (56.34)	1,296 (54.52)
	Yes	8,144 (67.09)	887 (73.00)	5,470 (43.66)	1,081 (45.48)

The statistical results and figures were processed with STATA statistical software version 14.0 (StataCorp LP, College Station 77845, USA) and Excel 2016, respectively. A two-tailed *p*-value of < 0.05 was considered statistically significant.

## Results

### Sample Characteristics

Table 2 presents the basic characteristics between the urban and rural participants before and after the implementation of the URRBMI policy. Of the 13,360 participants before the policy was introduced, 12,139 (90.90%) and 1,215 (9.10%) lived in rural and urban areas, respectively. After policy implementation, there were 12,529 (84.05%) rural participants and 2,377 (15.95%) urban participants. The average age of the persons sampled increased from 59.24 in 2011 to 61.91 in 2018. In general, the education level improved as the illiteracy rate decreased from 31.34 to 26.29%. The majority of the participants in all groups lived with others, slept more than 7 h, and did not smoke or drink. Only 19.25% of the participants had a disability, whereas 34.61% of them felt body pain prior to the policy; on the contrary, 17.71% of the participants had a disability, whereas 60.36% of them felt body pain after the introduction of the policy.

### Distribution of Chronic Diseases

For the rural participants, a greater number of females than males had chronic diseases (χ^2^ = 26.95, *p* < 0.001), and the prevalence of chronic diseases gradually increased with age (χ^2^ = 62.13, *p* < 0.001) and economic status (χ^2^ = 65.85, *p* < 0.001). Illiterate participants (χ^2^ = 86.05, *p* < 0.001), participants that living alone (χ^2^ = 5.50, *p* = 0.019), those sleeping <6 h (χ^2^ = 93.56, *p* < 0.001), those who smoke (χ^2^ = 127.45, *p* < 0.001), those who do not drink (χ^2^ = 77.04, *p* < 0.001), those with a disability (χ^2^ = 343.73, *p* < 0.001), and those with body pain (χ^2^ = 495.94, *p* < 0.001) were more likely to have chronic diseases (Table 3). Similarly, the urban participants' sex (χ^2^ = 5.39, *p* = 0.020), age (χ^2^ = 30.96, *p* < 0.001), and education level (χ^2^ = 20.38, *p* < 0.001) were significantly associated with the occurrence of chronic diseases. Additionally, participants sleeping <6 h (χ^2^ = 15.09, *p* = 0.001), those who smoke (χ^2^ = 23.15, *p* < 0.001), those who do not drink (χ^2^ = 10.26, *p* = 0.001), those with a disability (χ^2^ = 99.77, *p* < 0.001), and those with body pain (χ^2^ = 39.34, *p* < 0.001) were also more likely to have chronic diseases (Table 4).

**Table 3:** Distribution of chronic disease rate among rural participants (*n* = 24,668).

**Variables**	**Group**	**Chronic disease**		***N***	***P***
		**No**	**Yes**		
**Policy**	Before	3,995(32.91)	8,144(67.09)	12,139	<0.001
	After	7,059(56.34)	5,470(43.66)	12,529	
**Demographics**					
Sex	Male	5,367(46.58)	6,156(53.42)	11,523	<0.001
	Female	5,685(43.28)	7,450(56.72)	13,135	
Age (years)	45–50	2,055(48.67)	2,167(51.33)	4,222	<0.001
	51–60	3,960(46.30)	4,592(53.70)	8,552	
	61–70	3,161(42.21)	4,327(57.79)	7,488	
	≥71	1,878(42.62)	2,528(57.38)	4,406	
Education	Illiterate	3,131(41.33)	4,444(58.67)	7,575	<0.001
	≤ Elementary school	4,994(44.77)	6,161(55.23)	11,155	
	≥Middle school	2,923(49.33)	3,002(50.67)	5,925	
Economic status	Low	2,987(48.93)	3,118(51.07)	6,105	<0.001
	Middle	5,435(44.58)	6,757(55.42)	12,192	
	High	2,542(41.69)	3,555(58.31)	6,097	
**Life style**					
Living status	Live with others	8,795(45.20)	10,665(54.80)	19,460	0.019
	Live alone	2,259(43.38)	2,949(56.62)	5,208	
Sleeping hours	7–8 h	4,306(48.67)	4,541(51.33)	8,847	<0.001
	≤ 6 h	5,214(41.98)	7,205(58.02)	12,419	
	>8 h	1,534(45.09)	1,868(54.91)	3,402	
Nap	No	4,737(44.87)	5,819(55.13)	10,556	0.862
	Yes	6,317(44.76)	7,795(55.24)	14,112	
Smoking status	No	6,355(44.01)	8,086(55.99)	14,441	<0.001
	Yes	1,861(35.07)	3,445(64.93)	5,306	
Alcohol consumption	No	7,158(42.87)	9,538(57.13)	16,696	<0.001
	Yes	3,884(48.82)	4,072(51.18)	7,956	
**Health status**					
Disability	No	7,957(46.30)	9,229(53.70)	17,186	<0.001
	Yes	1,225(30.25)	2,824(69.75)	4,049	
Body pain	No	6,563(51.65)	6,143(48.35)	12,706	<0.001
	Yes	4,491(37.54)	7,471(62.46)	11,962	

**Table 4:** Distribution of chronic disease rate among urban participants (*n* = 3,592).

**Variables**	**Group**	**Chronic disease**		***N***	***P***
		**No**	**Yes**		
**Policy**	Before	328 (27.00)	887 (73.00)	1,215	<0.001
	After	1,296 (54.52)	45.07 (45.48)	2,377	
**Demographics**					
Sex	Male	736 (47.42)	816 (52.58)	1,552	0.020
	Female	888 (43.53)	1,152 (56.47)	2,040	
Age (years)	45–50	331 (52.79)	296 (47.21)	627	<0.001
	51–60	644 (47.18)	721 (52.82)	1,365	
	61–70	414 (40.04)	620 (59.96)	1,034	
	≥71	235 (41.52)	331 (58.48)	566	
Education	Illiterate	233 (43.07)	308 (56.93)	541	<0.001
	≤ Elementary school	612 (41.49)	863 (58.51)	1,475	
	≥Middle school	778 (49.40)	797 (50.60)	1,575	
Economic status	Low	287 (44.63)	356 (55.37)	643	0.062
	Middle	839 (47.11)	942 (52.89)	1,781	
	High	478 (42.68)	642 (57.32)	1,120	
**Life style**					
Living status	Live with others	1,245 (44.56)	1,549 (55.44)	2,794	0.142
	Live alone	379 (47.49)	419 (52.51)	798	
Sleeping hours	7–8 h	670 (48.91)	700 (51.09)	1,370	0.001
	≤ 6 h	795 (42.18)	1,090 (57.82)	1,885	
	>8 h	159 (47.18)	178 (52.82)	337	
Nap	No	621 (46.00)	729 (54.00)	1,350	0.461
	Yes	1,003 (44.74)	1,239 (55.26)	2,242	
Smoking status	No	1,004 (44.72)	1,241 (55.28)	2,245	<0.001
	Yes	194 (33.62)	383 (66.38)	577	
Alcohol consumption	No	1,065 (43.40)	1,389 (56.60)	2,454	0.001
	Yes	558 (49.12)	578 (50.88)	1,136	
**Health status**					
Disability	No	1,258 (47.65)	1,382 (52.35)	2,640	<0.001
	Yes	109 (22.95)	366 (77.05)	475	
Body pain	No	956 (50.10)	952 (49.90)	1,908	<0.001
	Yes	668 (39.67)	1,016 (60.33)	1,684	

### Effect of Policy on Health Outcomes

**Figure 2:**
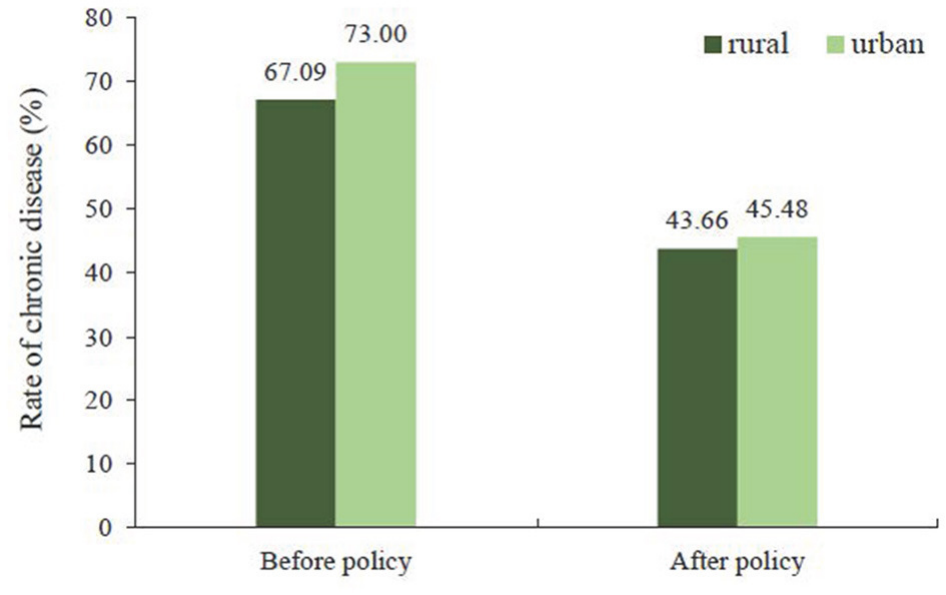
Rate of chronic disease for urban residents and rural residents.

Figure 2 shows that 67.09% (95% CI: 66.25, 67.93) of the rural participants had chronic diseases before the introduction of the policy and 43.66% (95% CI: 42.79, 44.53) after; meanwhile, 73.00% (95% CI: 70.41, 75.48) of the urban participants had chronic diseases before the introduction of the policy and 45.48% (95% CI: 43.46, 47.50) after.

Table 5 shows the multivariate analysis of the effect of the URRBMI policy on health outcomes by the GLMM. Compared to before the introduction of the policy in the urban population in model 1, the risk of having chronic diseases decreased by 70% (OR = 0.30; 95% CI: 0.26, 0.35) after the policy had been implemented; in the rural participants, the risk of having chronic diseases was 22% lower (OR = 0.78; 95% CI: 0.67, 0.90) than the risk in the urban participants prior to the policy and 71% lower (OR = 0.29; 95% CI: 0.25, 0.33) after. The differences in the ORs decreased from 0.21 prior to the policy to 0.01 after. By controlling for the demographic characteristics in model 2, the differences changed from 0.27 to 0.03; in model 3, the differences also tended to decrease after controlling for the factors in model 2 and adding lifestyle and health status characteristics. This suggests that the intervention of the URRBMI policy has had some positive effect on narrowing the gap in the chronic disease prevalence rates between the urban and rural populations.

**Table 5:** Effect of urban and rural residents' basic medical insurance policy on health outcome (*n* = 28,442).

**Variables**	**Model 1**				**Model 2**				**Model 3**			
	**OR**	**95%CI**		***P***	**OR**	**95%CI**		***P***	**OR**	**95%CI**		***P***
		**Lower**	**Upper**			**Lower**	**Upper**			**Lower**	**Upper**	
**Main variable**												
Before policy in urban	1.00				1.00				1.00			
Before policy in rural	0.78	0.67	0.90	0.001	0.73	0.63	0.85	<0.001	0.70	0.60	0.82	<0.001
*Difference_1_*	*0.21*	*-*	*-*	*-*	*0.27*	*-*	*-*	*-*	*0.30*	*-*	*-*	*-*
After policy in urban	0.30	0.26	0.35	<0.001	0.25	0.21	0.30	<0.001	0.19	0.16	0.23	<0.001
After policy in rural	0.29	0.25	0.33	<0.001	0.22	0.19	0.26	<0.001	0.16	0.14	0.19	<0.001
*Difference_2_*	*0.01*	*-*	*-*	*-*	*0.03*	*-*	*-*	*-*	*0.03*	*-*	*-*	*-*
**Control variables**												
Female					1.17	1.11	1.24	<0.001	1.02	0.93	1.12	0.678
Age (51–60 years)					1.32	1.22	1.42	<0.001	1.30	1.20	1.42	<0.001
Age (51–60 years)					1.74	1.61	1.89	<0.001	1.70	1.55	1.87	<0.001
Age (≥71 years)					1.75	1.60	1.92	<0.001	1.56	1.39	1.76	<0.001
≤ Elementary school					1.08	1.01	1.16	0.018	1.07	0.99	1.16	0.092
≥Middle school					0.97	0.90	1.05	0.476	1.02	0.92	1.12	0.757
Middle economic status					0.85	0.80	0.91	<0.001	0.90	0.82	0.98	0.017
High economic status					0.76	0.69	0.82	<0.001	0.88	0.79	0.98	0.018
Live alone									1.03	0.96	1.12	0.402
Sleeping ≤ 6 h									1.24	1.16	1.33	<0.001
Sleeping >8 h									0.94	0.85	1.04	0.233
Nap									1.11	1.04	1.19	0.002
No smoking									1.09	0.99	1.20	0.088
No alcohol consumption									0.80	0.74	0.87	<0.001
Disability									1.64	1.50	1.79	<0.001
Body pain									2.68	2.50	2.88	<0.001

*OR, Odds Ratios; CI, Confidence Interval; Difference_1_, The difference of ORs between urban and rural before policy; Difference_2_, The difference of ORs between urban and rural after policy; Model 1 only included policy and residence variable, Model 2 included variables in model 1 adding participants' sex, age, education, economics status, Model 3 included variables in model 1 adding participants' living status, sleeping hours, napping or not, smoking status, alcohol consumption, disability and body pain or not*.

Model 3 also shows that the prevalence of chronic diseases gradually increased with age and decreased with education (*p* <0.001). Those participants that sleep <6 h, that have naps, and that have a disability and body pain experienced increased prevalence of chronic diseases, while not drinking was shown to be a protective factor against chronic diseases.

## Discussion

The immediate function of health insurance is to guarantee the financial accessibility of health service utilization for vulnerable groups, with the ultimate goal of maintaining and improving health overall (23, 24). Chronic diseases, especially cardiovascular diseases, diabetes, cancer, and chronic respiratory diseases, are the leading cause of mortality across the world (25). Although previous studies have shown that social determinants may decrease the prevalence of chronic diseases, there is still a gap between urban and rural populations (26, 27). In this study, we focused on China's URRBMI policy and explored its effects on reducing the gap in the chronic disease prevalence between rural and urban populations when adjusting for their socioeconomic characteristics. According to these two representative middle-aged and elderly samples, the evidence supports the positive effect of the policy in terms of reducing the differences in the chronic disease prevalence rates between rural and urban populations, implied the positive effect of policy on progress in advancing UHC. This finding is consistent with the URRBMI policy evaluation studies by Liu et al., Yang et al., and Li et al. (15, 16, 28). It is also in line with the studies in Mexico (17), Vietnam (29), Peru (30), and Turkey (31) on the positive effect of health policy in achieving UHC.

These results provide evidence of the positive effect of integrating NRCMS and URBMI on improving the rural population's health outcomes. Now, the chronic disease prevalence for rural middle-aged and elderly populations is similar to that of urban middle-aged and elderly populations (43.66 vs. 45.48%). These findings also imply that the URRBMI policy plays an important role in moving China toward achieving UHC. There are two reasons that may explain this positive effect: First, according to previous studies, the adjustment of the coverage of health insurance affects the health service utilization of enrollees (32, 33). The URRBMI policy expanded the coverage of service packages and drug lists for rural enrollees, improving their access to health care and thus leading to improved health outcomes. Second, the literature suggests that higher health insurance reimbursement rates increase the financial accessibility of rural enrollees and promote greater utilization of health services (34, 35). The URRBMI policy in this study promotes UHC by increasing the reimbursement rate of medical expenses to promote timely access to health care for rural populations, which in turn improves their health outcomes.

Globally, chronic diseases affect the health and quality of life of populations (36). Therefore, the search for factors influencing the prevalence of chronic diseases never stops (26, 37, 38). This study found the prevalence of chronic diseases to gradually increase with age and to decrease with education; populations that sleep <6 h, that have naps, and that consume alcohol are more likely to experience an increased prevalence of chronic diseases, as are populations with a disability or body pain. Therefore, comprehensive prevention strategies and health promotion projects should be effectively strengthened in order to prevent an epidemic of chronic diseases.

### Limitations

We acknowledge that this study has several limitations that need to be addressed in further research. First, this study was based on two cross-sectional studies, and there may be some possible unobserved confounding factors, such as physical activity, BMI and work status. Second, the imbalanced data from rural (24,668) and urban (3,592) areas may have generated less statistically efficient results (e.g., larger standard error and less statistical significance) compared to more balanced data. Third, our study reports the effect of the policy on health outcomes based on quantitative research, and more evidence based on qualitative measurements and cohort studies on the analysis of the effects of the implementation of the policy is needed to clarify any cause–effect relationships in subsequent research steps. Last but not last, CHARLS only collected the data among middle-aged and elderly people; the adolescent and younger adults may have different health patterns.

## Conclusion

This paper shows that the basic medical insurance policy for urban and rural residents has a positive effect on narrowing the gap in the health outcomes between urban and rural residents in China, indicating that the integration of urban and rural medical insurance has promoted the realization of universal health coverage. The study also provides theoretical reference for the future development of health and the planning and formulation of health policies. However, more qualitative research and quantitative research based on evidence from cohort studies that include more determinants to analyze the effects of this policy is needed to clarify any causal relationships in the subsequent research steps.

## Data Availability Statement

The datasets and questionnaire are available at: http://charls.pku.edu.cn/.

## Ethics Statement

The studies involving human participants were reviewed and approved by Institutional Review Board of Peking University (protocol code IRB00001052-11015 and January 2011). The patients/participants provided their written informed consent to participate in this study.

## Author Contributions

MS was responsible for the conceptualization. MS and DW provided constructive suggestions on data analysis. XF, DW, and YZ were responsible for the sorting of data. XF and YZ did the statistical analysis. XF, YS, DW, and YZ was prepared by the manuscript. All authors read and approved the final manuscript.

## Funding

This research was funded by the National Natural Science Foundation of China (grant number: 72004178) and Natural Science Foundation of Inner Mongolia (grant number: 2020BS07002).

## Conflict of Interest

The authors declare that the research was conducted in the absence of any commercial or financial relationships that could be construed as a potential conflict of interest.

## Publisher's Note

All claims expressed in this article are solely those of the authors and do not necessarily represent those of their affiliated organizations, or those of the publisher, the editors and the reviewers. Any product that may be evaluated in this article, or claim that may be made by its manufacturer, is not guaranteed or endorsed by the publisher.

## References

[1] Verrecchia R Thompson R Yates R. Universal Health Coverage and public health: a truly sustainable approach. Lancet Public Health. (2019) 4:e10–1. 10.1016/S2468-2667(18)30264-0 30551975

[2] Jamison DT Alwan A Mock CN Nugent R Watkins D Adeyi O. Universal health coverage and intersectoral action for health: key messages from Disease Control Priorities, 3rd edition. Lancet. (2018) 391:1108–20. 10.1016/S0140-6736(17)32906-9 29179954 PMC5996988

[3] Bloom G Katsuma Y Rao KD Makimoto S Yin JDC Leung GM. Next steps towards universal health coverage call for global leadership. BMJ. (2019) 365:l2107. 10.1136/bmj.l2107 31126926 PMC6533546

[4] Tangcharoensathien V Mills A Patcharanarumol W Witthayapipopsakul W. Universal health coverage: time to deliver on political promises. Bull World Health Organ. (2020) 98:78. 10.2471/BLT.20.250597 32015572 PMC6986228

[5] Lin W Liu GG Chen G. The Urban Resident Basic Medical Insurance: a landmark reform towards universal coverage in China. Health Econ. (2009) 18(Suppl.2):S83–96. 10.1002/hec.1500 19551750

[6] Mao W Zhang Y Xu L Miao Z Dong D Tang S. Does health insurance impact health service utilization among older adults in urban China? A nationwide cross-sectional study. BMC Health Serv Res. (2020) 20:630. 10.1186/s12913-020-05489-8 32646423 PMC7346393

[7] Chen Q Chu X Wang S Zhang Zhang B A triple-difference approach to re-evaluating the impact of China's new cooperative medical scheme on incidences of chronic diseases among older adults in rural communities. Risk Manag Healthc Policy. (2020) 13:643–59. 10.2147/RMHP.S244021 32617028 PMC7325006

[8] Si W. Public health insurance and the labor market: evidence from China's Urban Resident Basic Medical Insurance. Health Econ. (2021) 30:403–31. 10.1002/hec.4198 33253447

[9] Pan J Tian S Zhou Q Han W. Benefit distribution of social health insurance: evidence from china's urban resident basic medical insurance. Health Policy Plan. (2016) 31:853–9. 10.1093/heapol/czv141 26936094

[10] Li X Zhang W. The impacts of health insurance on health care utilization among the older people in China. Soc Sci Med. (2013) 85:59–65. 10.1016/j.socscimed.2013.02.037 23540367

[11] Zhu K Zhang L Yuan S Zhang X Zhang Z. Health financing and integration of urban and rural residents' basic medical insurance systems in China. Int J Equity Health. (2017) 16:194. 10.1186/s12939-017-0690-z 29115955 PMC5678764

[12] Song X Zou G Chen W Han S Zou X Ling L. Health service utilisation of rural-to-urban migrants in Guangzhou, China: does employment status matter? Trop Med Int Health. (2017) 22:82–91. 10.1111/tmi.12801 27775826

[13] ext-link-type="uri" xlink:href="http://www,.gov.cn">www.gov.cn. Opinion on the Integration of Basic Medical Insurance Systems Between Urban Rural Residents. (2016). Available online at: http://www.gov.cn/zhengce/content/2016-01/12/content_10582.htm

[14] Yao Y Sun B Dong K. Annual Report on the Development of China's Ageing Finance (2016): Social Sciences Academic Press (CHINA). Singapore: Springer (2016).

[15] Yang X Chen M Du J Wang Z. The inequality of inpatient care net benefit under integration of urban-rural medical insurance systems in China. Int J Equity Health. (2018) 17:173. 10.1186/s12939-018-0891-0 30466451 PMC6251195

[16] Liu P Guo W Liu H Hua W Xiong L. The integration of urban and rural medical insurance to reduce the rural medical burden in China: a case study of a county in Baoji City. BMC Health Serv Res. (2018) 18:796. 10.1186/s12913-018-3611-y 30340575 PMC6195716

[17] Riumallo-Herl C Aguila E. The effect of old-age pensions on health care utilization patterns and insurance uptake in Mexico. BMJ Glob Health. (2019) 4:e001771. 10.1136/bmjgh-2019-001771 31798987 PMC6861075

[18] Ranabhat CL Jakovljevic M Kim CB Simkhada P. COVID-19 pandemic: an opportunity for universal health coverage. Front Public Health. (2021) 9:673542. 10.3389/fpubh.2021.673542 34395361 PMC8358071

[19] Ranabhat CL Kim CB Singh A Acharya D Pathak K Sharma B. Challenges and opportunities towards the road of universal health coverage (UHC) in Nepal: a systematic review. Arch Public Health. (2019) 77:5. 10.1186/s13690-019-0331-7 30740223 PMC6360747

[20] Zhao Y Hu Y Smith JP Strauss J Yang G. Cohort profile: the China Health and Retirement Longitudinal Study (CHARLS). Int J Epidemiol. (2014) 43:61–8. 10.1093/ije/dys203 23243115 PMC3937970

[21] Zhao Y Strauss J Yang G Giles J Hu PP Hu Y. China Health and Retirement Longitudinal Study, 2011-2012 National Baseline Users' Guide. Beijing: Peking University (2013).

[22] Wu Y Zhang L Liu X Ye T Wang Y. Geographic variation in health insurance benefits in Qianjiang District, China: a cross-sectional study. Int J Equity Health. (2018) 17:20. 10.1186/s12939-018-0730-3 29402292 PMC5800004

[23] Yuan B Jian W He L Wang B Balabanova D. The role of health system governance in strengthening the rural health insurance system in China. Int J Equity Health. (2017) 16:44. 10.1186/s12939-017-0542-x 28532418 PMC5440979

[24] Meng Q Yuan B Jia L Wang J Yu B Gao J. Expanding health insurance coverage in vulnerable groups: a systematic review of options. Health Policy Plan. (2011) 26:93–104. 10.1093/heapol/czq038 20813837

[25] Geneau R Stuckler D Stachenko S McKee M Ebrahim S Basu S. Raising the priority of preventing chronic diseases: a political process. Lancet. (2010) 376:1689–98. 10.1016/S0140-6736(10)61414-6 21074260

[26] Cockerham WC Hamby BW Oates GR. The social determinants of chronic disease. Am J Prev Med. (2017) 52:S5–S12. 10.1016/j.amepre.2016.09.010 27989293 PMC5328595

[27] Peng BL Ling L. Association between rural-to-urban migrants' social medical insurance, social integration and their medical return in China: a nationally representative cross-sectional data analysis. BMC Public Health. (2019) 19:86. 10.1186/s12889-019-6416-y 30658619 PMC6339269

[28] Li C Tang C Wang HA-O. Effects of health insurance integration on health care utilization and its equity among the mid-aged and elderly: evidence from China. Int J Equity Health. (2019) 18:166. 10.1186/s12939-019-1068-1 31665019 PMC6820904

[29] Nguyen BT Lo Sasso AT. The effect of universal health insurance for children in Vietnam. Health Econ Policy Law. (2019) 14:299–314. 10.1017/S1744133117000159 28482945

[30] Neelsen S O'Donnell O. Progressive universalism? The impact of targeted coverage on health care access and expenditures in Peru. Health Econ. (2017) 26:e179–203. 10.1002/hec.3492 28205370

[31] Atun R Aydin S Chakraborty S Sümer S Aran M Gürol I. Universal health coverage in Turkey: enhancement of equity. Lancet. (2013) 382:65–99. 10.1016/S0140-6736(13)61051-X 23810020

[32] Hao Y Wu Q Zhang Z Gao L Ning N Jiao M. The impact of different benefit packages of Medical Financial Assistance Scheme on health service utilization of poor population in rural China. BMC Health Serv Res. (2010) 10:170. 10.1186/1472-6963-10-170 20565720 PMC2909998

[33] Kim S Kwon S. The effect of extension of benefit coverage for cancer patients on health care utilization across different income groups in South Korea. Int J Health Care Finance Econ. (2014) 14:161–77. 10.1007/s10754-014-9144-y 24691773

[34] Li C Tang C Wang H. Investigating the association of health system characteristics and health care utilization: a multilevel model in China's ageing population. J Glob Health. (2020) 10:020802. 10.7189/jogh.10.020802 33312509 PMC7719298

[35] Miao Y Gu J Zhang L He R Sandeep S Wu J. Improving the performance of social health insurance system through increasing outpatient expenditure reimbursement ratio: a quasi-experimental evaluation study from rural China. Int J Equity Health. (2018) 17:89. 10.1186/s12939-018-0799-8 29940956 PMC6019724

[36] Bonilla-Sierra P Vargas-Martínez AM Davalos-Batallas V Leon-Larios F Lomas-Campos MD. Chronic diseases and associated factors among older adults in Loja, Ecuador. Int J Environ Res Public Health. (2020) 17:4009. 10.3390/ijerph17114009 32512938 PMC7312073

[37] Lyu J Zhang W Li W Wang S Zhang J. Epidemic of chronic diseases and the related healthy lifestyle interventions in rural areas of Shandong Province, China. BMC Public Health. (2020) 20:606. 10.1186/s12889-020-08729-y 32357867 PMC7195749

[38] Reiners F Sturm J Bouw LJW Wouters EJM. Sociodemographic factors influencing the use of eHealth in people with chronic diseases. Int J Environ Res Public Health. (2019) 16:645. 10.3390/ijerph16040645 30795623 PMC6406337

